# MYXOID NEUROFIBROMA: AN UNUSUAL PRESENTATION

**DOI:** 10.4103/0019-5154.39742

**Published:** 2008

**Authors:** Rosa Maria Ponce-Olivera, Andres Tirado-Sanchez, Amelia Peniche-Castellanos, Jorge Peniche-Rosado, Patricia Mercadillo-Perez

**Affiliations:** *From Service of Dermatology, Hospital General de México*; 1 *From Department of Dermatopathology, Hospital General de México*

**Keywords:** *Myxoid neurofibroma*, *nerve sheath tumor*, *neurofibroma*

## Abstract

Myxoid neurofibroma (MN) is a benign tumor of perineural cell origin, which is demonstrated with a positive immunohistochemical staining for S-100 protein. The most common locations of the MN are the face, shoulders, arms, periungual and in the feet. To our knowledge, this is the first time that a trunk location is reported. MN should be included in the differential diagnosis of tumors on this location.

## Introduction

Neurofibroma can present as a single lesion or be part of a neurofibromatosis.^1^ Solitary neurofibroma can present as one of the following variants: cutaneous lipomatous, collagenous, epithelioid, granular, pigmented, dendritic cell and myxoid neurofibromas.^2^

We present the case of a myxoid neurofibroma (MN) of the trunk, which is a rare location of this tumor.

## Case Report

**Fig. 1:**
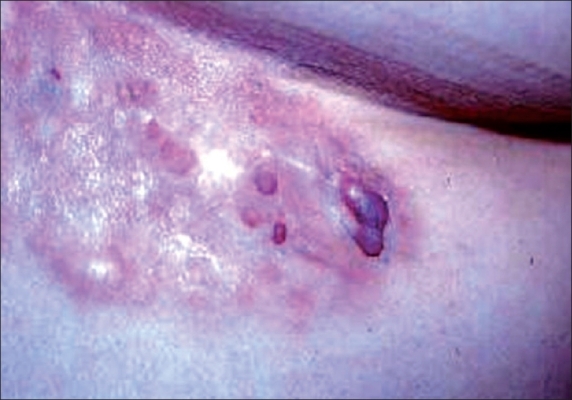
Skin-colored, slow-growing nodular plaque on the anterior trunk

A 44-year-old woman presented with a non-tender, slow-growing nodular plaque on the anterior trunk that appeared six years ago (Fig. 1). She sought medical attention for cosmetic reasons.

The patient denied any past medical condition or consumption of medication. There were no drug allergic reactions. On physical examination, a 10 × 5-cm flesh-colored plaque on the anterior trunk was observed.

**Fig. 2:**
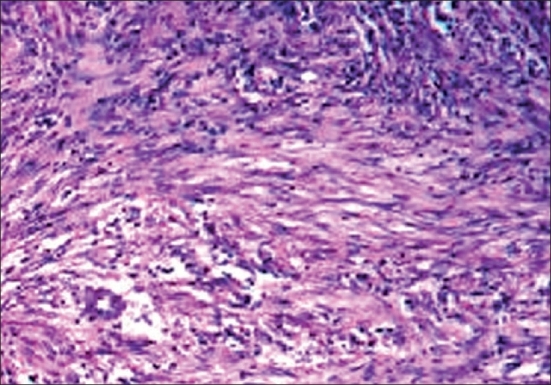
Numerous spindle-shaped cells with wavy nuclei and mucin (H and E, 40×)

An incisional biopsy was taken. Gross examination shows a well-circumscribed lesion composed of spindle-shaped cells with wavy nuclei (Fig. 2). A few mast cell and mucin were present. Immunohistochemical staining was positive for S-100 protein and negative for CD34. The diagnosis made was myxoid neurofibroma.

We scheduled a surgical excision that was incomplete and a few months later, a new lesion emerged, a new surgical procedure was programmed but the patient rejected it.

## Discussion

Myxoid neurofibroma is a benign tumor of perineural cell origin, which is demonstrated with a positive immunohistochemical staining for S-100 protein.^3^

This tumor has a higher incidence in young adults.^4^ It usually presents as a non-tender solitary nodule.^3^

The most common locations of the MN are the face, shoulders, arms, periungual and in the feet.^5^,^6^ To our knowledge, this is the first time that a trunk location has been reported.

MN is usually a solitary lesion,^1^ as in our patient, however, they can be numerous and may recur following an incomplete initial excision.

Differential diagnosis includes intramuscular myxoma and myxoid dermatofibrosarcoma protuberans. The first one is negative to S-100; and in the second one, on histological examination, slender tumor cells with large, spindle-shaped nuclei are seen, as well as mitotic figures; a high cellularity and irregular, short, intersecting bands of tumor cells forming a storiform pattern are characteristic.^7^,^8^

The usual first-line treatment is total excision of tumor,^9^ but only for cosmetic or diagnostic reasons. Some reports have mentioned the danger of these tumors masquerading as malignancies (mainly neurofibrosarcoma or epithelioid sarcoma).^6^

To our knowledge, this is the first report of a MN on the trunk. MN should be included in the differential diagnosis of tumors at this location.
